# Meteorological Table

**Published:** 1857-09

**Authors:** J. G. Westmoreland

**Affiliations:** Atlanta, Ga.


### METEOROLOGICAL OBSERVATIONS FOR AUGUST, 1857, AT ATLANTA, GA.

| AUGUST. | THERMOMETER. | | | BAROMETER. | | | WIND. | REMARKS. |
|---|---|---|---|---|---|---|---|---|
| | 7 A. M. | 2 P. M. | 7 P. M. | 7 A. M. | 2 P. M. | 7 P. M. | | |
| 1 | 71 | 80 | 77 | 30. | 30. | 30. | S. | Cloudy—Rain 1. |
| 2 | 72 | 80 | 72 | 30. | 30. | 30. | S. | Cloudy—Rain ¼. |
| 3 | 70 | 76 | 72 | 30. | 30.05 | 30.05 | S. W. | Cloudy—Rain ⅞. |
| 4 | 72 | 70 | 70 | 30.07 | 30.07 | 30.07 | S. W. | Cloudy—Rain ½. |
| 5 | 74 | 84 | 78 | 30. | 30. | 30. | S. W. | Hazy. |
| 6 | 70 | 86 | 72 | 30. | 30.05 | 30.05 | W. | Fair. |
| 7 | 70 | 88 | 78 | 30.10 | 30.15 | 30.20 | E. | Hazy. |
| 8 | 72 | 84 | 78 | 30.25 | 30.25 | 30.27 | E. | Hazy. |
| 9 | 66 | 86 | 80 | 30.17 | 30.20 | 30.12 | N. E. | Fair. |
| 10 | 62 | 84 | 72 | 30.05 | 30. | 29.95 | E. | Fair. |
| 11 | 74 | 84 | 76 | 30. | 30. | 30.05 | S. W. | Cloudy—Rain ¼. |
| 12 | 72 | 90 | 78 | 30.15 | 30.20 | 30.20 | W. | Hazy. |
| 13 | 72 | 90 | 90 | 30.25 | 30.22 | 30.27 | S. W. | Fair. |
| 14 | 82 | 92 | 80 | 30.30 | 30.27 | 30.27 | W. | Fair. |
| 15 | 86 | 94 | 84 | 30.25 | 30.20 | 30.15 | W. | Fair. |
| 16 | 86 | 94 | 90 | 30.25 | 30.25 | 30.20 | W. | Fair. |
| 17 | 78 | 92 | 88 | 30.22 | 30.25 | 30.17 | W. | Fair. |
| 18 | 78 | 92 | 87 | 30.20 | 30.20 | 30.12 | W. | Fair. |
| 19 | 76 | 84 | 70 | 30.12 | 30.10 | 30.07 | S. W. | Cloudy—Rain ⅛. |
| 20 | 66 | 78 | 66 | 30.10 | 30.10 | 30.10 | E. | Cloudy. |
| 21 | 64 | 78 | 66 | 30.12 | 30.12 | 30.10 | N. W. | Fair. |
| 22 | 64 | 88 | 70 | 30.05 | 30. | 30. | W. | Hazy. |
| 23 | 66 | 82 | 70 | 30.02 | 30.02 | 30. | N. | Fair. |
| 24 | 60 | 80 | 74 | 30. | 30.15 | 30.15 | N W. | Fair |
| 25 | 64 | 76 | 70 | 30.20 | 30.25 | 30.25 | N. | Hazy. |
| 26 | 80 | 86 | 80 | 30.27 | 30.15 | 30.15 | W. | Cloudy—Rain ¼. |
| 27 | 72 | 86 | 80 | 30.10 | 30.10 | 30.05 | S. | Hazy. |
| 28 | 74 | 86 | 78 | 30.05 | 30. | 30. | S. W. | Fair. |
| 29 | 58 | 62 | 70 | 30. | 30.12 | 30. | S. W. | Fair. |
| 30 | 58 | 78 | 68 | 30.05 | 30.15 | 30.15 | W. | Fair. |
| 31 | 64 | 82 | 74 | 30.15 | 30.25 | 30.20 | W. | Fair. |

Furnished by

J. G. WESTMORELAND, M. D.

